# Impact of buffered sodium butyrate as a partial or total dietary alternative to lincomycin on performance, *IGF*-1 and *TLR*4 genes expression, serum indices, intestinal histomorphometry, *Clostridia*, and litter hygiene of broiler chickens

**DOI:** 10.1186/s13028-023-00704-y

**Published:** 2023-09-28

**Authors:** Basma Mohamed Bawish, Mohamed Farahat Selem Zahran, Elshaimaa Ismael, Shaimaa Kamel, Yasmine H. Ahmed, Dalia Hamza, Taha Attia, Khaled Nasr Eldin Fahmy

**Affiliations:** 1 Department of Veterinary Hygiene and Management, Faculty of Veterinary Medicine, Cairo University, PO Box 12211, Giza, 12211 Egypt; 2 Department of Pharmacology, Faculty of Veterinary Medicine, University of Sadat City, Minoufiya, 23897 Egypt; 3 Department of Biochemistry and Molecular Biology, Faculty of Veterinary Medicine, Cairo University, Giza, 12211 Egypt; 4 Department of Cytology and Histology, Faculty of Veterinary Medicine, Cairo University, Giza, 12211 Egypt; 5 Department of Zoonoses, Faculty of Veterinary Medicine, Cairo University, Giza, 12211 Egypt; 6 Department of Nutrition and Clinical Nutrition, Faculty of Veterinary Medicine, Cairo University, Giza, 12211 Egypt

**Keywords:** Butirex C4®, Caecal *Clostridia*, Dressing yield, Lincomix® 50, Litter nitrogen, Liver function

## Abstract

**Background:**

Sodium butyrate (SB) is a short-chain fatty acid and a safe antibiotic alternative. During 35 days, this study compared the impact of coated SB (Butirex C4) and lincomycin (Lincomix) on broiler growth, gut health, and litter hygiene in 1200 one-day-old Ross-308 broiler chicks that were randomly assigned into 5-dietary groups with 5-replications each. Groups divided as follows: T1: Basal diet (control), T2: Basal diet with buffered SB (1 kg/ton starter feed, 0.5 kg/ton grower-finisher feeds), T3: Basal diet with 100 g/ton lincomycin, T4: Basal diet with buffered SB (0.5 kg/ton starter feed, 0.25 kg/ton grower-finisher feeds) + 50 g/ton lincomycin, and T5: Basal diet with buffered SB (1 kg/ton starter feed, 0.5 kg/ton grower-finisher feeds) + 50 g/ton lincomycin. Birds were housed in a semi-closed deep litter house, where feed and water were available ad libitum. Results were statistically analyzed using ANOVA and Tukey’s post hoc tests.

**Results:**

Combined dietary supplementation with SB and lincomycin (T4 and T5) significantly enhanced body weights, weight gains, feed conversion ratio, and profitability index. Also, carcasses in T4 and T5 exhibited the highest dressing, breast, thigh, and liver yields. T5 revealed the best blood biochemical indices, while T3 showed significantly elevated liver and kidney function indices. T4 and T5 exhibited the highest expression levels of *IGF-1* and *TLR4* genes, the greatest villi length of the intestinal mucosa, and the lowest levels of litter moisture and nitrogen. *Clostridia perfringens* type A alpha-toxin gene was confirmed in birds’ caeca, with the lowest clostridial counts defined in T4.

**Conclusions:**

Replacing half the dose of lincomycin (50 g/ton) with 0.5 or 1 kg/ton coated SB as a dietary supplement mixture showed the most efficient privileges concerning birds’ performance and health.

## Background

Over the past decades, using antibiotics in subtherapeutic doses as feed additives for animal and poultry diets has achieved multiple benefits of promoting growth, minimizing mortalities, and reducing production costs although, numerous safety worries emerged due to that practice, as the persistent inclusion of antibiotics in the food animal industry caused injury to animal’s intestines, suppression of gut health, contamination of animal products with antibiotic residues, and polluting of the environment with antibiotic-resistant bacteria [1, 2]. All these drawbacks challenged animal producers to find efficient growth-promoting substitutes for antibiotics.

The gut microbiome is a critical determinant of a bird’s performance and productivity [3], and recent investigations reported the disturbance effects of antibiotics on intestinal microbiota [1, 4]. Tang et al. [1] administered lincomycin (1 g/kg feed) to weaned piglets (21 days of age) for one week and documented adverse health effects due to the lowered level of intestinal beneficial bacteria confronted by the rise in potential pathogens and decreased body weights. The same findings, in addition to immunosuppression, were reported by Zhang et al. [5] following the administration of lincomycin to young mice for one week in drinking water (1 g/L water). Lincomycin did not influence feed efficiency, broiler immunity, or intestinal microbial counts in the Azeem trial [6].

Lincomycin is a natural lincosamide antibiotic that originates from *Streptomyces lincolnensis* and is usually used against intestinal gram-positive bacterial infections. Lincomycin absorption through the gastrointestinal tract (GIT) was reported to be very weak, and its primary side effect is gastrointestinal inflammation, especially with long-term usage [5, 7]. The lowered body weight gain of animals that received lincomycin was attributed to its negative impact on intestinal permeability, wall thickness, and nutrient absorbability [6].

Short-chain fatty acids (SCFAs) are vital metabolites in the intestinal microbiome and are essential as anti-inflammatory agents and for supporting gut health. In the Romick et al. [8] and Zhang et al. [5] studies, lincomycin was reported to destroy numerous intestinal SCFA-producing bacteria and lower the production of SCFAs and butyrate. SCFA and their salts are generally considered safe alternatives to antibiotics for animals. Poultry producers widely applied butyric acid in broiler chicken diets as an alternative feed additive to antibiotic growth promotors (AGP). SB is the sodium salt of butyric acid characterized by its stability, non-odorous, and ease of handling during feed manufacturing [9, 10]. SB is supplemented in either free form to encourage the upper GIT development or buffered (coated) to prevent its dissociation in the upper part of GIT and enhance its bioavailability in lower GIT; due to its slow release [11].

The buffered SB promotes intestinal mucosa modulation, regulates gene expression, augments SCFAs production, and improves protein synthesis [12–14]. SB activates the expression of Toll-like receptors 4 (*TLR4*) and insulin-like growth factor-1 (*IGF*-1) but decreases DNA fragmentation induced by pathogenic bacteria like *Clostridium perfringens* [15–17]. SB has an antimicrobial effect by increasing the synthesis of mucin and antimicrobial peptides and decreasing intestinal epithelial permeability [18]. Moreover, it favours intestinal health by increasing the viable counts of *Lactobacillus* and *Bifidobacterium* beneficial bacteria [19]. SB is an energy source for enterocytes resulting in increased intestinal villi development and absorptive surface [12, 20, 21]. SB possesses anti-inflammatory, antioxidant and immune-enhancing properties [22–25], as it enhances protein digestibility and serves as a substrate in intermediate metabolism, thus improving the broiler’s overall health and performance [26].

Previous studies indicated the application of coated SB at a rate of 600 to 1000 mg/kg of feed could enhance broiler chicken weights and feed conversion rates [2, 10]. Further investigations reported that no significant body weight differences were obtained [13]. Yet, most of the experiments proved the modulating effect of coated SB long-term administration on gut microbiota concerning promoting the beneficial microbes [2, 13].

The current study examined the efficiency of dietary buffered SB 54% (Butirex C4®) supplement to replace lincomycin (Lincomix® 50) addition to broiler feed entirely or partly. The experimental design tested varying dosage regimens of both products. The evaluation included measuring a range of indices involving the weekly and overall performance parameters, carcass traits, blood biochemistry, intestinal histology and microbial counts, tissue gene expressions (*IGF*-1 and *TLR4*), and litter microbial and chemical conditions.

## Methods

### Experimental design, diets, and housing

The Institutional Animal Care and Use Committee, Faculty of Veterinary Medicine, Cairo University, Egypt, approved the experimental design (Vet CU 12/10/2021/347). The trial was conducted at the Animal and Poultry Research Center, Faculty of Veterinary Medicine, Cairo University, Giza, Egypt.

A total of 1200 one-day-old Ross-308 broiler chicks from a commercial hatchery were weighed and randomly allocated into five groups with five replicates each (n = 45 birds/replicate). The bird grouping was as follows: T1; received basal diet (control), T2; basal diet with Butirex C4 (1 kg/ton in starter feed (0–15 d); and 0.5 kg/ton in grower and finisher feeds (16–35 d)), T3; basal diet with Lincomix at a level of 100 g/ton (the recommended dose) from 0 to 35 d, T4; basal diet with 50% of Butirex C4 (0.5 kg/ton at 0–15 d, and 0.25 kg/ton at 16–35 d) and a half dose of Lincomix (50 g/ton), and T5; basal diet with Butirex C4 (1 kg/ton at 0–15 d, and 0.5 kg/ton at 16–35 d) and a half dose of Lincomix (50 g/ton).

Butirex C4® (Novation, Spain) is a novel feed additive of 54% SB coated with a physicalchemical matrix of buffer salts. Lincomix® 50 (Zoetis Services LLC., USA) is a growth promotor and broad-spectrum antibiotic (lincomycin hydrochloride). The basal corn-soya bean meal-based diet was formulated to meet the nutrient requirements of Ross 308 broilers [27] during the starter (1–15 d), grower (16–28 d), and finisher (29–35 d) phases (Table 1).

**Table 1:** Physical and chemical compositions of basal diets for each growing period

Items	Starter (0 to 14 days)	Grower (15 to 28 days)	Finisher (29 to 35 days)
**Ingredients %**			
Yellow corn	55.24	59.39	63.64
Soybean meal 46%	27.00	18.60	10.30
Full fat SBM	8.00	12.50	16.00
Corn gluten meal 60%	6.00	6.00	6.50
Monocalcium phosphate	0.90	0.80	0.80
Limestone	1.60	1.50	1.50
NaCl	0.35	0.35	0.35
Sod. bicarbonate	0.10	0.10	0.10
L-Lysine	0.25	0.25	0.30
DL-Methionine	0.15	0.10	0.10
Toxin binder	0.10	0.10	0.10
Quantum blue (Phytase)	0.01	0.01	0.01
Broiler premix^1^	0.30	0.30	0.30
**Chemical analysis**:			
ME (Kcal/kg)	3001.05	3100.63	3200.24
Crude Protein (%)	23.17	21.11	19.14
Crude Fat (%)	3.96	4.87	5.60
Calcium (%)	1.00	0.94	0.93
P. Available (%)	0.50	0.45	0.42

^1^Vitamin and mineral mixture contained: 13,000,000 IU vitamin A; 6,000,000 IU vitamin D3; 80,000 mg vitamin E; 4000 mg vitamin K; 5000 mg vitamin B1; 9000 mg vitamin B2; 5000 mg vitamin B6; 35 mg vitamin B12; 20,000 mg pantothenic acid; 70,000 mg Nicotinic acid; 2000 mg Folic acid; 250 mg Biotin; 400,000 mg choline chloride; 120,000 mg Manganese oxide; 100,000 mg Zinc oxide; 15,000 mg Copper sulphate; 1000 calcium Iodide; 50,000 mg ferrous sulphate; 350 mg Selenium selenite

The house with a semi-closed ventilation system was subdivided into 25 identical floor pens of 2.9 m × 1.6 m dimensions. The floor type was concrete covered with 7–10 cm wood shaving litter. Birds received similar managemental and hygienic conditions. After the chicks had arrived at the poultry house, they received 24 h of light for the first three days, then maintained under 23 L:1D for the remainder of the experiment. The temperature of the house was 32 ± 1 °C for the first 3-days, then gradually reduced by 0.5 °C per day until it declined to 24 °C [28]. The humidity ranged between 55 and 60% throughout the experimental period [29]. Clean water was available *ad libitum* in bell-shaped drinkers (4-litre capacity) and replaced with 8-litre drinkers in older age [30]. Feed was available ad libitum via round plastic feeders throughout the experimental period [31].

Broilers received Newcastle disease virus (NDV) Hitchner B1 vaccination on the day 6. While on day 18, birds received NDV-Lasota vaccination through intraocular administration. Infectious Bursal Disease (IBD) and Avian Influenza (H5N1) vaccines were administered on day 14 (0.2 mL/bird) through S/C injection [32].

### Growth parameters

Body weights (BW) of broilers were recorded weekly for each replicate on a pen basis at 1, 7, 14, 21, 28, and 35 days of age. The weekly body weight gain (BWG), average weekly feed intake (FI) and feed conversion ratio (FCR) for each week and the overall period of the experiment (from 1 to 35 days of age) was measured, as described previously [32]. Daily bird mortalities were recorded for each group. After adjusting mortality, FCR was calculated by dividing weekly feed intake by weekly weight gain. At the end of the experiment, EPEF (European Production Efficiency Factors) was calculated according to the following formula [29]: EPEF = (livability (%) × live body weight (kg) / (age in days × FCR) × 100.

### Carcass characteristics and immune organs

At the end of the experiment (day 35), 25 birds (5 birds/treatment) were slaughtered, defeathered, and eviscerated after 12 h of fasting [33]. Carcasses were weighted after removing the head, neck, and legs. Then, carcasses were dissected to measure the relative weights of breast, thigh, and drumstick muscles [34]. Moreover, the giblet weights (gizzard, liver (without gall bladder) and heart) and immune organ weights (spleen and bursa of Fabricius) were recorded and expressed as a percentage of live weight [35].

### Blood biochemical analysis

At the end of the experiment (day 35), five blood samples per bird group were collected from the jugular vein after slaughter. After centrifuging for 15 min at 3000 rpm, sera were separated and stored at -20 °C until analysis. In serum samples, different biochemical parameters were measured by spectrophotometer (UV-2100 Spectrophotometer, USA) using spectrum diagnostics kits (Spectrum Diagnostics Egyptian Company for Biotechnology). Serum albumin [36], total protein [37], triglycerides [38], cholesterol [39], uric acid [40], alanine transaminase (ALT), and aspartate transaminase (AST) [41] were determined according to the manufacturer’s instructions.

### NDV vaccinal antibody titers

Haemagglutination inhibition test was performed to assess NDV antibody titers, following [42] guidance. Two-fold serial dilutions of 25 µL of each serum sample were conducted in 99-V-bottomed microwell plates. To each well, 25 µL of four haemagglutination units of ND-Lasota commercial antigen were added, and plates were incubated at room temperature for 20 min. To each well, 25 µL of 1% chicken-RBC suspension was added. Antibody titers were reported as mean log_2_ haemagglutination inhibition titers.

### Gene expression analysis

About 100 mg of breast and thigh muscles and liver tissue were dissected from five birds per group. The samples were disrupted in a lysis buffer solution using a tissue homogenizer. Samples were processed for total RNA extraction according to the protocol of the easy-spin Total RNA Extraction Kit (Cat. No.17,221; iNtRON Biotechnology DR). The quantity and purity of RNA were assessed using Nanodrop [43]. The cDNA synthesis was performed using M-MLV Reverse Transcriptase (enzynomics Cat. # RT001S). The transcript level of *IGF-1* (growth-related gene) in both breast and thigh muscles and *TLR4* (immune response-related gene) in the liver were evaluated at the mRNA level by qRT-PCR using RealMOD™ Green W² 2x qPCR mix (Cat. No 25,350) according to the manufacturer’s instructions. Each RT-PCR was performed in triplicate [44]. Real-time quantitation of mRNAs was normalized to an endogenous reference of the *β-actin* gene [45]. The fold change was calculated by the comparative threshold cycle (C_T_) method (2^−ΔΔCt^) [46]. The primers used in Real-time PCR were designed using Primer 3 program (https://primer3.ut.ee/) [47–49]. The primers’ sequences were shown in Table 2.

**Table 2:** Primers sequences used for qRT-PCR

Gene symbol	Gene description	Accession number	Primer Sequence
*IGF1*	Insulin Like Growth Factor 1	NM_001004384.2	F: 5′-ACTGTGTGGTGCTGAGCTGGTT-3′ R: 5′-AGCGTGCAGATTTAGGTGGCTT-3′
*β-actin*	Beta-actin	L08165.1	F:- 5′-CCCACACCCCTGTGATGAAA-3′ R:- 5′-TAGAACTTTGGGGGCGTTCG-3′
*TLR-4*	Toll-like receptor 4	NM_001030693.1	F: 5′-ATGTCCTCTTGCCATCCCAA-3′ R: 5′-TCTCCCCTTTCTGCAGAGTG-3′

### Microbiological examination of caecal content and deep litter

After birds were slaughtered at day 35, five caecal contents and five deep-litter samples were collected from each group for microbiological examination. The upper 7 cm of deep litter were scraped from 3 different spots of each replicate pen and placed in sterile plastic bags [50, 51]. All samples were maintained at 4 °C until examination.

For microbial examination of caecal content, a 10^− 1^ dilution was prepared by diluting and homogenizing 1 g of each sample in 9 ml sterile saline solution, followed by 10-fold serial dilutions till the 10^− 10^ dilution [52]. For microbial examination of litter samples, 3 g of each homogenized litter sample were transferred to tubes containing 27 mL sterile saline solution (10^− 1^ dilution). Samples were kept at room temperature for 30–60 min and frequently shacked to allow the litter to mix well with the diluent [50, 51], followed by 10-fold serial dilutions till the 10^− 14^ dilution. Then, 100 µl were taken from the last 3-dilutions and spread onto Nutrient Agar (Hi-Media Laboratories, India) plates to enumerate total aerobes and incubated for 24–48 h at 37 °C. Another 100 µL were spread onto Reinforced Clostridial Agar (Oxoid Ltd, Basingstoke, Hants, UK) plates to enumerate total *Clostridia* and incubated for 24–48 h at 37 °C under anaerobic conditions. Finally, the counts of bacterial colonies were reported as mean 10-logarithm colony-forming units (log_10_ CFU) for each gram of litter and caecal content.

### Confirmation and toxin typing of *Clostridium perfringens*

Genomic DNA of suspected *C. perfringens* isolates was extracted using an extraction kit (QIA amp mini kit, Qiagen, Hilden, Germany). The multiplex PCR assay was used to detect the presence of genes encoding alpha-toxin (*cpa*), beta-toxin (*cpb*), epsilon-toxin (*etx*), iotatoxin (*iap*) and CPE (*cpe*) [53]. Primer sequences were published previously [53]. The PCR reaction mixtures were analyzed by electrophoresis on a 1.5% (w/v) agarose gel in the presence of a 100 bp DNA ladder (Fermentas Life Science, USA).

### Physical and chemical examinations of litter

The upper 7 cm of deep litter were scraped from 3 different spots of each replicate pen and placed in sterile plastic bags [50, 51]. Litter moisture was estimated by drying 10 g of litter samples in the hot air oven at 100 ± 5 °C for 24–48 h [50]. Moisture % was calculated by subtracting dry weights from the initial weights. Additionally, the total nitrogen content of litter samples was determined as total Kjeldahl nitrogen [54].

### Histomorphometric analysis

The duodenum, jejunum, and ileum from five birds per group were collected and flushed with saline solution (0.9% NaCl) to remove contents, then fixed in 10% neutral buffered formalin for 48 h for histological examination. After fixation, samples were dehydrated in ascending grades of ethyl alcohol, cleared in xylene, and embedded in paraffin wax. Sections of 3–4 μm in thickness were obtained by rotatory microtome, deparaffinized, and stained with haematoxylin and eosin (H&E) stain for examination under the light microscope [55].

H&E stained sections were used for the histomorphometry. Approximately five intestinal tissue sections were measured by a high-power lens (X 40). Parameters measured include villus height from the tip of the villus to the crypt and crypt depth from the base of the villi to the submucosa. A computerized microscopic image analyzer, attached to a full HD microscopic camera (Leica Microsystems, Germany), was used to determine the histomorphometric parameters using statistical analysis.

### Statistical analysis

Data were checked for normality by the Shapiro-Wilk test. Then, the mean differences were compared between groups via analysis of variance (ANOVA) and Tukey’s post hoc tests using PASW Statistics 18.0 software (SPSS Inc., Chicago, IL, USA). Results were reported as means and standard error of the mean (SEM). Charts were generated with R (Version 3.6.1, R Foundation for Statistical Computing) using ggplot2 [56], ggpubr [57], tidyverse [58], and rstatix [59] packages. The significance was considered at P < 0.05.

## Results

### Performance parameters

The results of the growth performance analysis (Table 3) revealed that no significant difference was noted between the mean values of BW, BWG, and FCR among the groups during the starter period (0–14 d). However, there was a significant decrease (P < 0.05) in day 14’s FI of T5 (1 kg/ton buffered SB combined with a half dose of lincomycin) compared to the control (T1). In addition, there was a significant increase (P < 0.05) in BW at the grower (15–28 d) and finisher (29–35 d) periods in T5, followed by T4. Moreover, during the finisher period, also there was an improvement in BW in T2. However, the lincomycin-supplemented group (T3) and control (T1) showed the lowest final BW. On day 35, and for the overall period, there was an improvement (P < 0.05) in BWG and FCR in T5, T4, and T2. Weekly FI of different groups at different periods did not differ statistically (P > 0.05) from each other. The highest cumulative feed intake was noted in T3, while the lowest was noted in T5. The EPEF (Table 4) was significantly (P < 0.05) the highest in all SB-supplemented groups (T2, T4, and T5) and lowered in lincomycin supplemented group (T3) and control (T1). The lowest mortality rate was recorded for T2, while the highest was in T3. However, mortality was not significantly (P > 0.05) different between bird groups.

**Table 3:** Influence of dietary sodium butyrate and lincomycin on weekly growth performance parameters of broiler chickens

		Body weight (g)						Weight gain (g)						Feed intake (g)						FCR (g/g)				
Groups		D 7	D 14	D 21	D 28	D 35		D 7	D 14	D 21	D 28	D 35		D 7	D 14	D 21	D 28	D 35		D 7	D 14	D 21	D 28	D 35
T1		193	526	1033^bc^	1700^b^	2157^bc^		149	332	507	667	457^b^		161	409^a^	716	1028	1114		1.08	1.23	1.41	1.54	2.47^ac^
T2		192	520	1013^c^	1691^b^	2228^abc^		149	327	494	678	537^a^		161	406^ab^	712	1018	1124		1.09	1.24	1.44	1.50	2.13^abc^
T3		193	524	1028^bc^	1695^b^	2142^c^		149	331	504	667	447^b^		160	405^ab^	718	1032	1129		1.07	1.22	1.43	1.55	2.53^a^
T4		191	523	1040^ab^	1717^ab^	2259^ab^		147	332	517	677	542^a^		164	405^ab^	716	1022	1022		1.11	1.22	1.39	1.51	2.08^bc^
T5		190	531	1055^a^	1729^a^	2269^a^		146	340	524	674	540^a^		163	398^b^	709	1011	1081		1.12	1.17	1.35	1.50	2.00^b^
SEM^1^		1.14	2.30	4.19	4.78	14.60		1.14	2.23	4.05	4.90	14.19		0.53	1.23	2.83	3.36	14.02		0.01	0.01	0.01	0.01	0.06
P-value		0.920	0.670	0.017	0.044	0.003		0.920	0.467	0.136	0.936	0.038		0.141	0.05	0.732	0.318	0.854		0.510	0.117	0.227	0.554	0.003

^a,b,c^ Mean values with different superscripts in the same column indicate significant difference (Tukey’s test; P ≤ 0.05)

T1: Control - basal diet; T2: Butirex C4 1 kg/ton in starter feed (0-15d) and then 0.5 kg/ton in grower and finisher feed; T3: Lincomix recommended dose (100 g/ton); T4: Lincomix half the recommended dose (50 g/ton) + Butirex C4 0.5 kg/ton at 0-15d and then 0.25 kg/ton; T5: Lincomix half the recommended dose (50 g/ton) + Butirex C4 1 kg/ton at 0-15d and then 0.5 kg/ton

FCR, Feed Conversion Ratio (g of feed / g of weight gain)

^1^ SEM: Standard error of mean

Number of sampled birds (N) = 20 birds/replicate (100 birds/group)

**Table 4:** Influence of dietary sodium butyrate and lincomycin on cumulative growth performance parameters of broiler chickens (days 1–35)

Groups	Body weight (g)	Weight gain (g)	Feed intake (g)	FCR (g/g)	EPEF	Mortality (%)
T1	2157^bc^	2113^bc^	3429	1.62^ac^	362^bc^	5.83
T2	2228^abc^	2184^abc^	3421	1.57^abc^	390^ab^	4.17
T3	2142^c^	2098^c^	3443	1.64^a^	354^c^	6.25
T4	2259^ab^	2215^ab^	3427	1.55^bc^	383^ab^	5.83
T5	2269^a^	2225^a^	3362	1.51^b^	392^a^	5.42
SEM^1^	14.60	14.60	15.79	0.01	5.02	0.48
P-value	0.003	0.003	0.565	0.001	0.035	0.726

^a,b,c^ Mean values with different superscripts in the same column indicate significant difference (Tukey’s test; P ≤ 0.05)

T1: Control - basal diet; T2: Butirex C4 1 kg/ton in starter feed (0-15d) and then 0.5 kg/ton in grower and finisher feed; T3: Lincomix recommended dose (100 g/ton); T4: Lincomix half the recommended dose (50 g/ton) + Butirex C4 0.5 kg/ton at 0–15 d and then 0.25 kg/ton; T5: Lincomix half the recommended dose (50 g/ton) + Butirex C4 1 kg/ton at 0–15 d and then 0.5 kg/ton

FCR, Feed Conversion Ratio (g of feed / g of weight gain)

EPEF: European Production Efficiency Factor= (livability × live weight (kg) / (age in days × FCR) × 100

^1^ SEM: Standard error of mean

Number of sampled birds (N) = 20 birds/replicate (100 birds/group)

### Carcass characteristics and immune organs

The inclusion of dietary buffered SB supplement with reducing lincomycin to half its dose (T4 and T5) showed a significant increase (P < 0.05) in dressing yield (Table 5). T4 exhibited the highest dressing yield, while the control (T1) showed the lowest value. The relative weight of drumstick muscle showed a significant decrease (P < 0.05) in control (T1) compared with other groups. T4 and T5 reported the highest breast %, while T5 reported the highest thigh% compared to other groups. Although, no significant differences (P > 0.05) were indicated in breast and thigh muscle percentages among all groups.

**Table 5:** Influence of dietary sodium butyrate and lincomycin on carcass characteristics and immune organs of broiler chickens (day 35)

Groups	Dressing (%)	Breast (%)	Thigh (%)	Drum (%)	Liver (%)	Gizzard (%)	Heart (%)	Spleen (%)	Bursa (%)
T1	71.76^c^	23.79	19.08	9.81^b^	2.71^ab^	1.15	0.57	0.14	0.23
T2	74.03^bc^	23.66	19.82	10.61^a^	2.87^ab^	1.04	0.58	0.15	0.22
T3	74.17^bc^	23.83	20.51	10.63^a^	2.56^b^	1.14	0.56	0.14	0.22
T4	77.13^a^	25.43	20.37	10.35^a^	2.98^a^	1.00	0.55	0.14	0.22
T5	74.61^ab^	24.32	20.83	10.30^a^	3.00^a^	1.10	0.60	0.14	0.21
SEM^1^	0.43	0.29	0.22	0.08	0.05	0.03	0.01	0.01	0.01
P- value	0.0001	0.280	0.086	0.0001	0.016	0.447	0.734	0.976	0.948

^a,b,c^ Mean values with different superscripts in the same column indicate significant difference (Tukey’s test; P ≤ 0.05)

T1: Control - basal diet; T2: Butirex C4 1 kg/ton in starter feed (0-15d) and then 0.5 kg/ton in grower and finisher feed; T3: Lincomix recommended dose (100 g/ton); T4: Lincomix half the recommended dose (50 g/ton) + Butirex C4 0.5 kg/ton at 0-15d and then 0.25 kg/ton; T5: Lincomix half the recommended dose (50 g/ton) + Butirex C4 1 kg/ton at 0-15d and then 0.5 kg/ton

^1^ SEM: Standard error of mean

Number of sampled birds (N) = 5 birds/group

There was a significant increase (P < 0.05) in the relative weight of the liver in groups that received SB and lincomycin combination (T5, T4), whereas; T3 reported significantly (P < 0.05) lowered liver %. No significant differences (P > 0.05) were observed in the relative weight of the gizzard, heart, and immune organs weights (spleen and Bursa of Fabricius) between different groups.

### Blood biochemical parameters

The highest serum total protein and albumin concentrations were shown in T5 (P < 0.05), as presented in Table 6) T2 reported the lowest serum cholesterol level but was not significantly different from other groups. The TAG concentration significantly decreased in all supplemented groups compared to control T1 (P < 0.05). ALT activity significantly dropped in T5 compared to other groups (P < 0.05). AST activity significantly decreased in T2 and T5 (P < 0.05). The uric acid concentration reported the lowest value in T5 (P < 0.05) and the highest in T1 and T3. In general, T5 showed the most satisfactory blood biochemical parameters, while T3 reported the worst liver and kidney function indices.

**Table 6:** Influence of dietary sodium butyrate and lincomycin on blood biochemical parameters of broiler chickens (day 35)

	Protein profile			Lipid profile			Liver functions		Kidney function
Groups	Total protein (g\dl)	Albumin (g\dl)		Cholesterol (mg\dl)	TAG (mg\dl)		ALT (U\L)	AST (U\L)	Uric acid (mg\dl)
T1	2.58^b^	1.44^ab^		102.76	113.06^a^		18.02^a^	254.02^a^	8.62^a^
T2	2.60^b^	1.22^b^		86.10	59.76^b^		17.42^a^	174.83^b^	6.70^a^
T3	2.74^b^	1.48^ab^		102.76	51.00^b^		20.99^a^	227.21^a^	7.12^a^
T4	2.96^ab^	1.28^b^		98.40	69.82^b^		17.13^a^	214.64^ab^	6.60^a^
T5	3.52^a^	1.68^a^		110.86	77.24^b^		11.27^b^	179.46^b^	3.52^b^
SEM^1^	0.10	0.05		15.58	5.13		0.86	7.24	2.09
P-value	0.005	0.0001		0.139	0.0001		0.002	0.0001	0.0001

^a,b^ Mean values with different superscripts in the same column indicate significant difference (Tukey’s test; P ≤ 0.05)

T1: Control - basal diet; T2: Butirex C4 1 kg/ton in starter feed (0–15 d) and then 0.5 kg/ton in grower and finisher feed; T3: Lincomix recommended dose (100 g/ton); T4: Lincomix half the recommended dose (50 g/ton) + Butirex C4 0.5 kg/ton at 0–15 d and then 0.25 kg/ton; T5: Lincomix half the recommended dose (50 g/ton) + Butirex C4 1 kg/ton at 0–15 d and then 0.5 kg/ton

TAG: Triacylglyceride, ALT: Alanine transaminase. AST: Aspartate transaminase

^1^ SEM: Standard error of mean

Number of sampled birds (N) = 5 birds/group

### Haemagglutination inhibition antibody titers of NDV

Haemagglutination inhibition titers of NDV antibodies in birds’ sera, at day 35, showed that T3, T4, and T5 presented higher mean haemagglutination inhibition titers (3.63, 2.50, and 2.58 log_2_, respectively) than T1 and T2 (2.25 and 1.20 log_2_, respectively). However, those levels did not exhibit significant differences (P = 0.430).

### *IGF-1* and *TLR4* genes expression

**Fig. 1:**
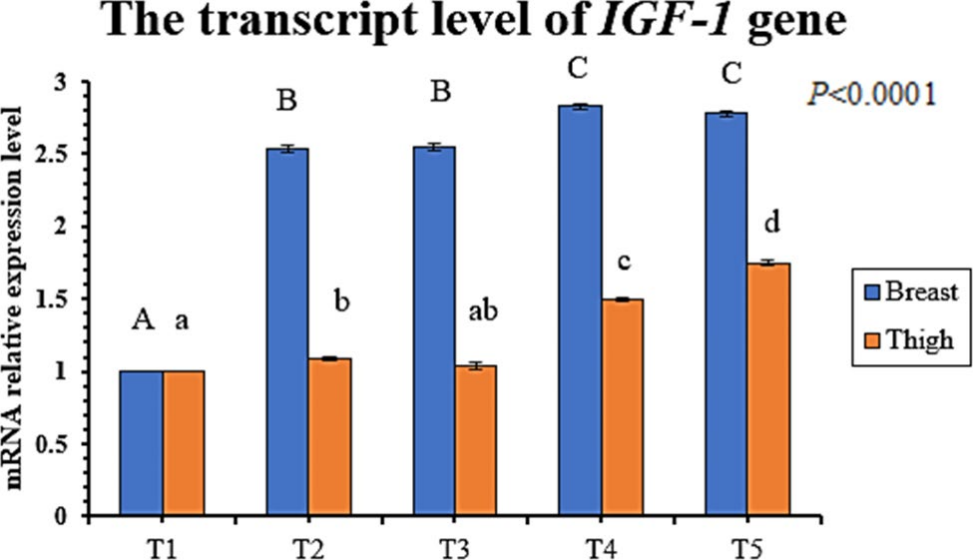
Influence of dietary sodium butyrate and lincomycin on mRNA relative expression level of *IGF* gene (growth-related gene) in both breast and thigh muscles of broiler chickens. Data are represented as mean ± SEM. Groups having different letters are significantly different from each other at P ≤ 0.05. Groups having similar letters are non-significantly different from each other at P ≤ 0.05. T1: Control - basal diet; T2: Butirex C4 1 kg/ton in starter feed (0–15 d) and then 0.5 kg/ton in grower and finisher feed; T3: Lincomix recommended dose (100 g/ton); T4: Lincomix half the recommended dose (50 g/ton) + Butirex C4 0.5 kg/ton at 0–15 d and then 0.25 kg/ton; T5: Lincomix half the recommended dose (50 g/ton) + Butirex C4 1 kg/ton at 0–15 d and then 0.5 kg/ton. Number of sampled birds (N) = 5 birds/group

**Fig. 2:**
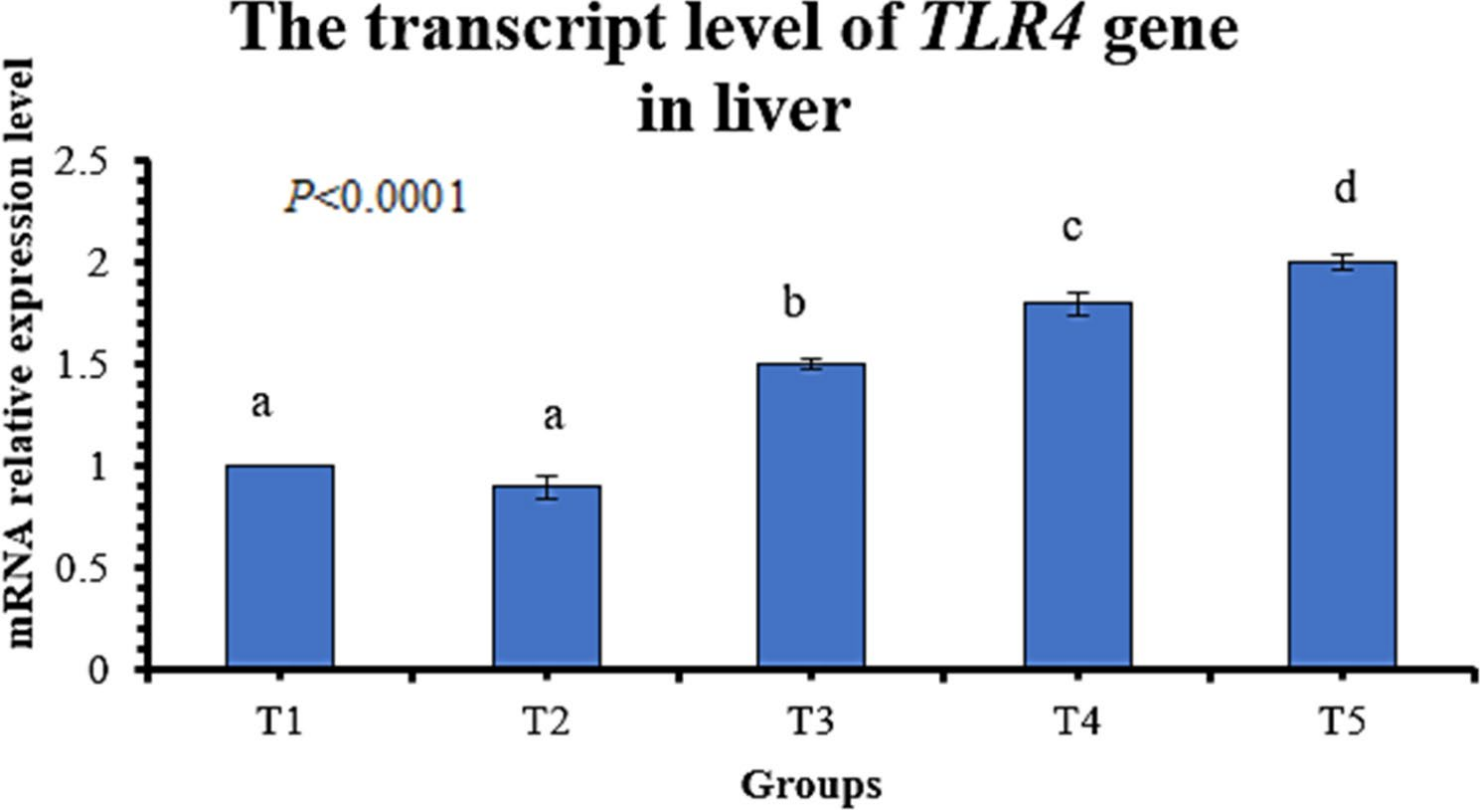
Influence of dietary sodium butyrate and lincomycin on mRNA relative expression level of *TLR4* gene in the liver of broiler chickens. Data are represented as mean ± SEM. Groups having different letters are significantly different from each other at P ≤ 0.05. Groups having similar letters are non-significantly different from each other at P ≤ 0.05. T1: Control - basal diet; T2: Butirex C4 1 kg/ton in starter feed (0–15 d) and then 0.5 kg/ton in grower and finisher feed; T3: Lincomix recommended dose (100 g/ton); T4: Lincomix half the recommended dose (50 g/ton) + Butirex C4 0.5 kg/ton at 0–15 d and then 0.25 kg/ton; T5: Lincomix half the recommended dose (50 g/ton) + Butirex C4 1 kg/ton at 0–15 d and then 0.5 kg/ton. Number of sampled birds (N) = 5 birds/group

Dietary SB, lincomycin, and their combined supplementation significantly (P < 0.05) increased the transcript level of the *IGF-1* gene (growth-related gene) in the breast muscle compared to the control (Fig. 1). The transcript level of the *IGF-1* gene in thigh muscle increased significantly in T2, T4, and T5 compared to control (T1). The transcript level of the *TLR4* gene (immune response-related gene) in the liver was significantly higher in T3, T4, and T5 than in T1 (Fig. 2). *IGF-1* and *TLR4* genes exhibited the highest expression levels in T4 and T5.

### Microbiological examination of caecal content

**Fig. 3:**
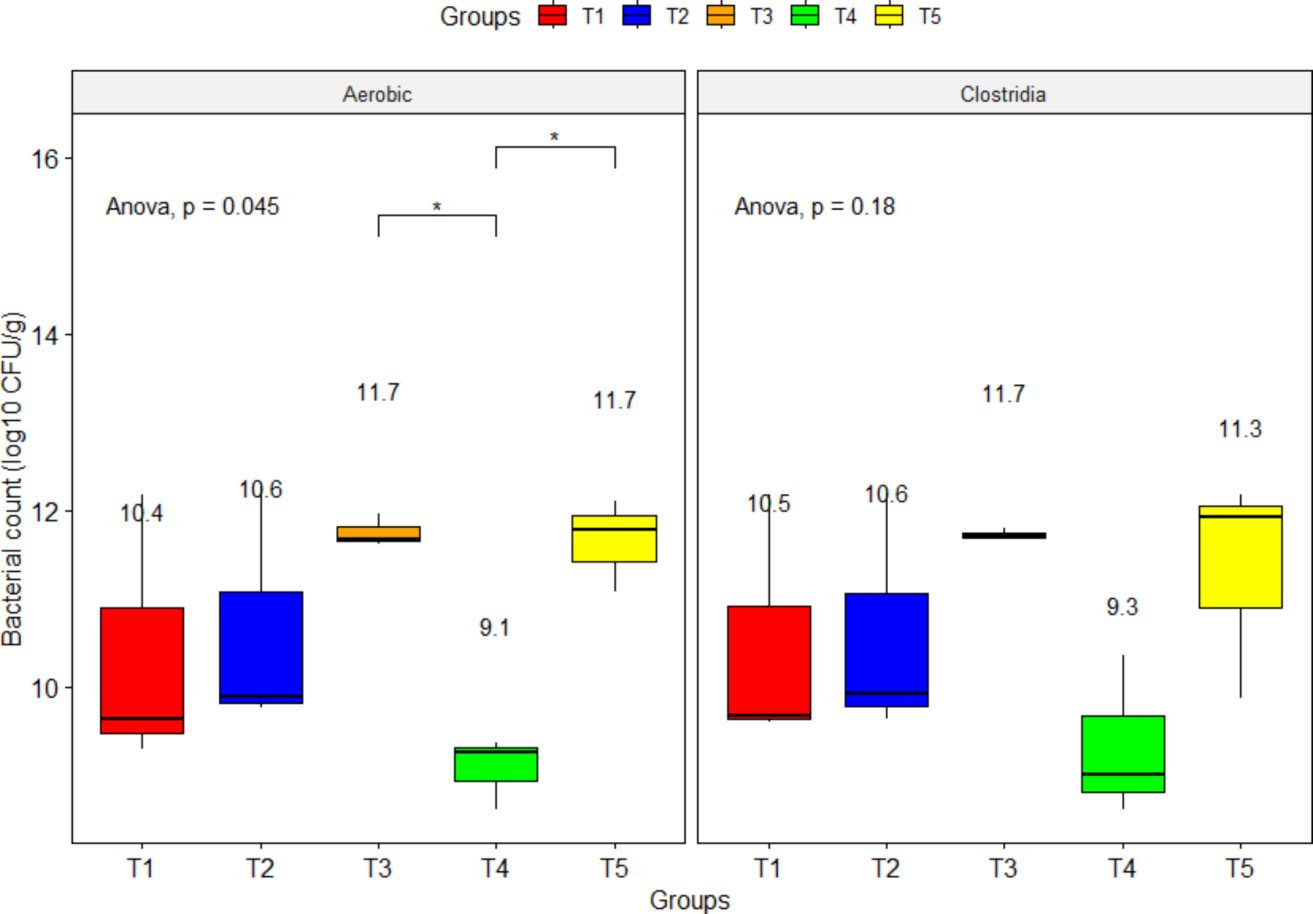
Influence of dietary sodium butyrate and lincomycin on caecal bacterial counts of broiler chickens (day 35). T1: Control - basal diet; T2: Butirex C4 1 kg/mt in starter feed (0–15 d) and then 0.500 kg/mt in grower and finisher feed; T3: Lincomix recommended dose (100 g) without Butirex C4; T4: Lincomix half the recommended dose (50 g) + Butirex C4 0.5 kg/mt at 0-15d and then 0.250 kg/mt; T5: Lincomix half the recommended dose (50 g) + Butirex C4 1 kg/mt at 0–15 d and then 0.500 kg/mt. Data shown above boxplots represent means. * Asterisks indicate significance at P ≤ 0.05. Number of sampled birds (N) = 5 birds/group

The bacterial counts per gram of caecal content were the lowest in the T4 bird group, with an average of 9.07 ± 0.24 log_10_ CFU of total aerobic bacteria (P = 0.045) and 9.32 ± 0.53 log_10_ CFU of total *Clostridia*. The T3 and T5 groups did not demonstrate improvement from the control group (T1) but had bacterial counts higher by nearly 1.19 log_10_ CFU/g (Fig. 3).

### Confirmation and toxin typing of *Clostridium perfringens*

The amplification of the *C. perfringens* type A alpha-toxin gene at 324 kb was confirmed in the suspected *C. perfringens* isolates of all groups. *C. perfringens* isolates from the control group (T1) displayed both *C. perfringens* type A alpha toxin and *C. perfringens* enterotoxin genes.

### Litter chemical and microbiological parameters

**Fig. 4:**
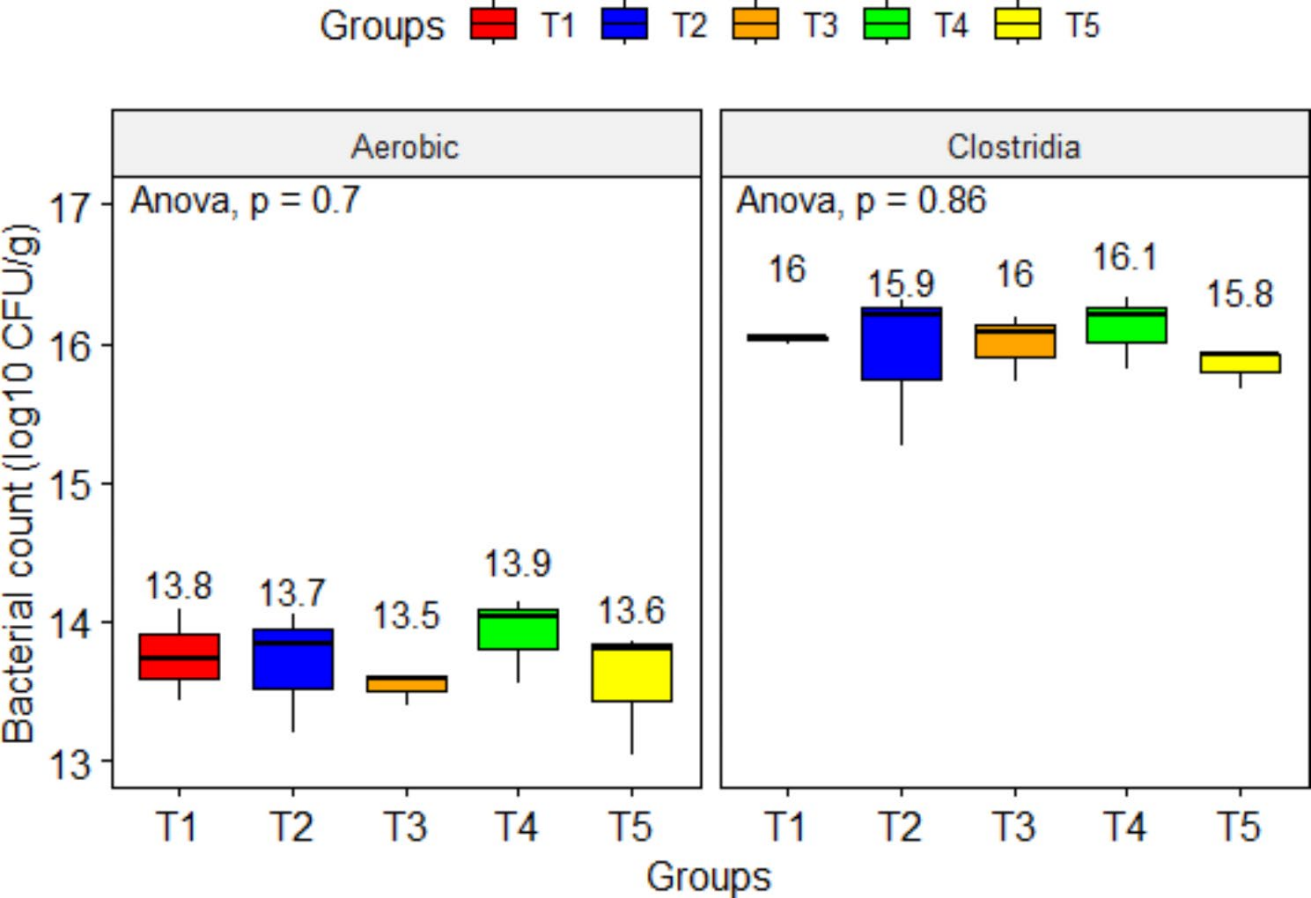
Influence of dietary sodium butyrate and lincomycin on deep litter bacterial counts of broiler chickens (day 35). T1: Control - basal diet; T2: Butirex C4 1 kg/mt in starter feed (0–15 d) and then 0.500 kg/mt in grower and finisher feed; T3: Lincomix recommended dose (100 g) without Butirex C4; T4: Lincomix half the recommended dose (50 g) + Butirex C4 0.5 kg/mt at 0–15 d and then 0.250 kg/mt; T5: Lincomix half the recommended dose (50 g) + Butirex C4 1 kg/mt at 0–15 d and then 0.500 kg/mt. Data shown above boxplots represent means. Significance was set at P ≤ 0.05. Number of litter samples (N) = 5 litter samples/group

Regarding the microbial quality of deep litter at day 35 (Fig. 4), there were no significant differences between the experimental groups (P > 0.05). Litter quality in different groups showed variable results. Moisture content (g/kg) recorded the highest levels for litter collected from T1 (323.41 ± 27.46) and T2 (323.25 ± 16.07), followed by T3 (318.16 ± 38.26) and T5 (300.41 ± 37.18), and the lowest value was for T4 (278.87 ± 32.90). However, these differences were not significant (P = 0.825). The nitrogen content of pooled litter samples displayed the lowest levels in T4 (3.63 g/kg), T5 (3.81 g/kg), and T3 (4.18 g/kg) groups compared to T1 (6.47 g/kg) and T2 (7.01 g/kg), with an average difference of -3.87 g/kg of litter.

### Histomorphometry of small intestine

**Fig. 5:**
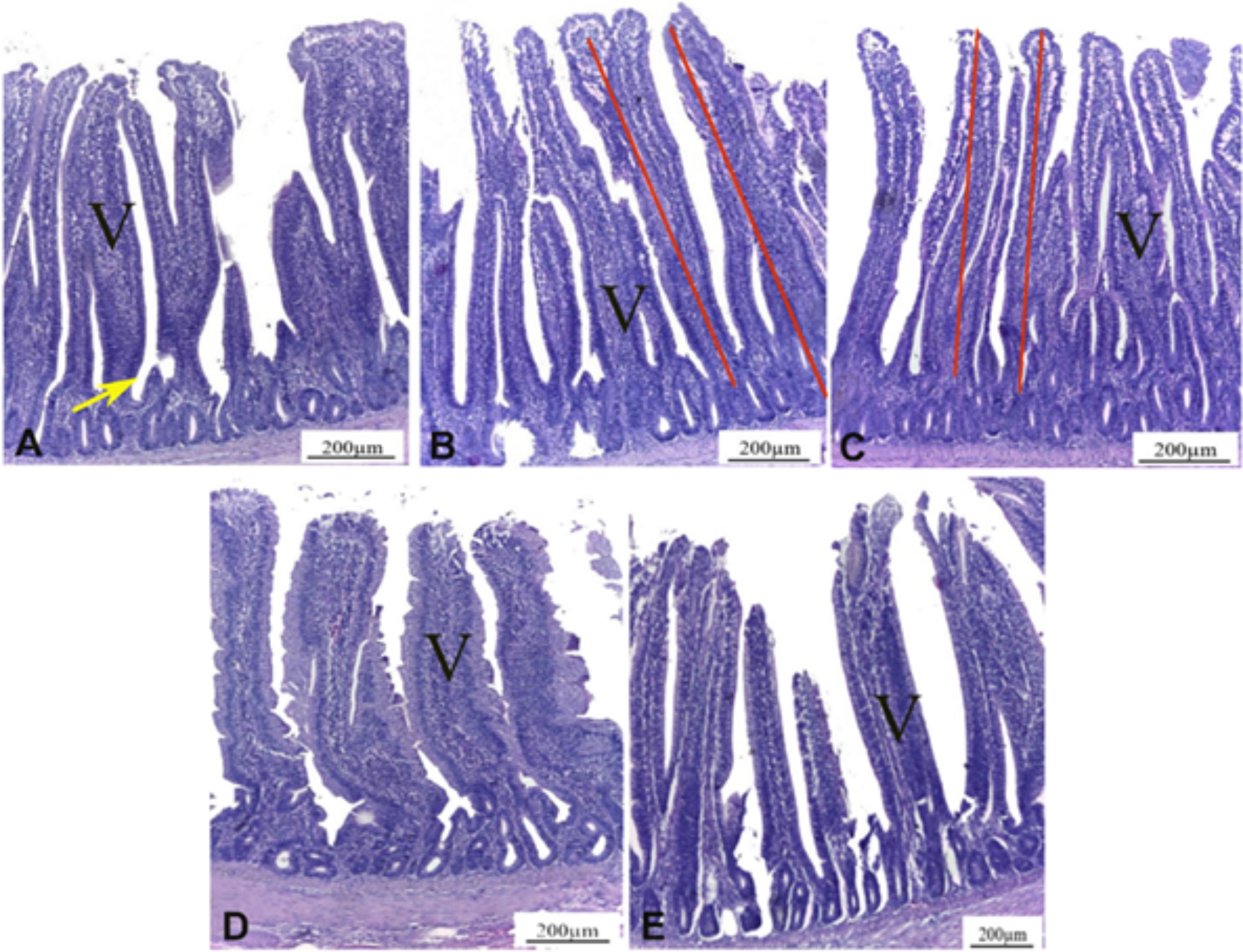
Influence of dietary sodium butyrate and lincomycin on intestinal histomorphometry of broiler chickens (day 35) Photomicrograph of jejunum. H&E. **V**: intestinal villi, **Arrow**: intestinal crypt and **Lines**: intestinal length. **A**: The control group showing the intestinal villi (V) and intestinal crypts (arrow). **B**: The Butirex (1 kg) fed group showing significant increase in the intestinal villi length (red lines) compared to control group. **C**: The Lincomix (100 g) fed group revealing significant increase in the length of villi (red lines) compared to control group. **D**: The Lincomix (50 g) plus Butirex (0.5 kg then 0.250 kg) fed group showing increase in the length of intestinal villi but not significant compared to groups 1, 2, and 3. **E**: The Lincomix (50 g) plus Butirex (1 kg then 0.5 kg) fed group exhibiting increase in the length of intestinal villi but not significant compared to groups 1, 2, 3, and 4. Number of sampled birds (N) = 5 birds/group

In the duodenum, the intestinal villi and crypt depth in T2, T3, T4 and T5 were significantly greater than in control group T1 (P < 0.05). There were significantly higher increases in the length of duodenal villi and crypt depth in T4 and T5 compared to T2 and T3. In the jejunum, T5 showed the highest villi length, followed by T4, while T3 exhibited the lowest values ((P < 0.05). Jejunal crypt depth was the largest in T5. In the ileum, T5 recorded the highest villi length and crypt depth, followed by T4 (P < 0.05) (Table 7; Fig. 5).

**Table 7:** Influence of dietary sodium butyrate and lincomycin on histomorphometry of the small intestine of broiler chickens (mean µm ± SE)

		Duodenum				Jejunum				Ileum		
Groups		Villi length	Crypt depth	C/V		Villi length	Crypt depth	C/V		Villi length	Crypt depth	C/V
T1		833^c^	81^c^	10.2		817^c^	89	9.1		757^c^	80^b^	9.4
T2		1100^b^	104^b^	10.0		957^c^	116	8.2		910^b^	91^b^	10.0
T3		1067^b^	99^bc^	10.1		813^c^	123	6.6		930^b^	101^b^	9.2
T4		1200^ab^	100^bc^	12.0		1100^b^	120	6.0		1077^ab^	118^b^	9.1
T5		1433^a^	121^a^	11.0		1337^a^	132	10.0		1233^a^	131^a^	9.4
SEM^1^		65.20	3.40	0.40		58.90	3.80	0.60		52.30	4.80	0.33
P-value		0.020	0.050	0.836		0.0001	0.400	0.529		0.030	0.020	0.982

^a,b,c^ Mean values with different superscripts in the same column indicate significant difference (Tukey’s test; P ≤ 0.05)

T1: Control - basal diet; T2: Butirex C4 1 kg/ton in starter feed (0–15 d) and then 0.5 kg/ton in grower and finisher feed; T3: Lincomix recommended dose (100 g/ton); T4: Lincomix half the recommended dose (50 g/ton) + Butirex C4 0.5 kg/ton at 0–15 d and then 0.25 kg/ton; T5: Lincomix half the recommended dose (50 g/ton) + Butirex C4 1 kg/ton at 0–15 d and then 0.5 kg/ton

C/V: Crypt/Villus ratio

^1^ SEM: Standard error of mean

Number of sampled birds (N) = 5 birds/group

## Discussion

In the current study, performance data during the starter period revealed that BW, BWG, and FCR were not affected by either the dietary supplementation of SB or lincomycin, which, according to an earlier study [60], may be explained by the immature digestive functionality of young birds at the starting phase. Birds are still developing their gut microbiota during the first week of life since it has not yet stabilized. As a result, large concentrations of some additives can disrupt the natural development of microflora, thus impacting bird performance [61]. Similarly, some studies documented that dietary supplementation of SB at increasing doses of 500, 1000, or 2000 mg/kg had no effect on broiler chicken growth performance during the starter phase [13, 62]. Other studies reported that AGP did not influence BW, FCR, and BWG during the starter period of broilers [63, 64]. Additional studies have shown that providing lincomycin as AGP to broilers benefited their growth rate [65–67]. However, in the current study, no significant differences were noticed in growth performance and feed efficiency between lincomycin and the control, as formerly observed [68]. This result may be due to increased feed intake, so it doesn’t imply greater feed conversion efficiency.

The combined supplementation of SB and lincomycin in T5 significantly reduced weekly FI at day 14. Other researchers have published similar findings; regarding the strong tendency of combined butyrate supplements to reduce feed intake and improve feed efficiency [69]. In grower and finisher periods, the partial or total SB dietary substitution to lincomycin in T5, T4, and T2 displayed an improvement in BW, BWG, FCR and the production profitability, which was indicated through the significant increase of EPEF as reported previously [70, 71]. This improvement could be due to the improved nutritional digestibility by SB that enhanced intestinal structure, as it augmented both crypt depth and villus height of the three intestinal segments, so raised intestinal absorption as indicated in our histomorphometric results. Butyrate has numerous beneficial effects on intestinal tissues and gut health as it is an essential energy source for the development and proliferation of the gastrointestinal epithelium [72, 73].

SB promotes nutrient digestibility and absorption due to the modification of gut microstructure and increased digestive enzymes’ activity, hence improving the productive efficiency of broilers [74, 75]. SB positively impacts protein and mineral digestibility, which boosts weight gain and FCR [76, 77]. Interestingly, SB beneficially modulates Toll-like receptor 4 (*TLR*4) activation by lowering the activation of mitogen-activated protein kinase and nuclear factor B (NF-B) pathways, as well as the generation of proinflammatory cytokines [15], so improves immune status and growth rate. Cumulative mortality was not significantly affected by SB or lincomycin supplementations. This result complies with previous research that showed no influence on the whole mortality rates by the graded levels of encapsulated butyric acids [63, 78] or lincomycin [79].

In the present study, SB and lincomycin supplements enhanced dressing yield, especially in T4. Similar studies showed that organic acids and antibiotic combination supplements exhibited higher carcass yields [80]. Furthermore, previous research [81] demonstrated that birds fed diets supplemented with butyric acid gained higher dressing yields when compared to other groups. Contrarily, in a recent study [82], no significant effects were observed on carcass traits through butyric acid supplementation compared to the control and antibiotic-supplemented groups. These findings may be attributed to elevated expression levels of insulin-like growth factors (*IGF*-1), which promote growth and enhances feed utilization efficiency in broilers [83], resulting in enhanced final live body weights and dressing yields.

According to our results, no significant differences were observed in breast and thigh muscles between different groups. However, the percentage of the economically valuable parts, like drumstick muscle, was increased in SB and lincomycin-supplemented groups compared to the control, which could indicate faster maturity due to rapid growth. Like our findings, drumstick meat yields increased by organic acid supplementation compared to the control group [84]. On the other hand, dietary butyric acid did not affect breast and thigh yields [85].

Combining dietary SB with lincomycin in T4 and T5 enhanced birds’ liver weights better than those supplemented with lincomycin (T3). That may be related to enhanced liver function indices via decreased ALT and AST levels in our biochemical results, as increased serum levels of AST demonstrated possible hepatocyte and liver dysfunction [86]. Similar studies reported a slight increase in the relative liver weight in birds fed organic acid-supplemented diets compared to Enramycin-supplemented diets [87]. No significant differences were observed in relative gizzard, heart, and immune organs weights. Likewise, heart and immune organs weights were not affected by the coated organic acids or antibiotic growth promoters [87, 88].

In this study, the mixture of SB and lincomycin in T5 significantly improved serum total protein concentrations, which indicated increased dietary protein utilization [32]. The increased proteolytic enzyme activity stimulates nutrient digestibility and elevates the absorption rate caused by increased intestinal villi length [89]. For the lipid profile, supplementary SB and lincomycin significantly lowered serum TAG in all treated groups. Similar trials suggested that butyrate influences gene expression, which regulates the catabolism of lipids [11]. The intake of animal meat products with low lipid content is beneficial for human health [90]. Also, SB supplementation in T2 and its combination with lincomycin in T4 and T5 improved the liver and kidney function indices. Previous findings [10] suggested that SB supplementation significantly decreased ALT and AST levels compared with the control group. Alike, serum uric acid levels were reduced in the butyric acid-supplemented groups [82]. Another study [91] reported that SB supplementation didn’t affect the serum levels of total protein, protein fractions, ALT, AST, and uric acid while decreasing the total and LDL cholesterol.

Haemagglutination inhibition antibody titer is a marker of birds’ humoral immunity. In this study, the SB and lincomycin supplements did not enhance the humoral immunity of broilers at 35 days of age against NDV compared to the control. An earlier study [10] indicated that SB supplement at application rates of 0.3, 0.6, and 1.2 g/kg of feed at different ages (14, 21, and 28 days) could raise the humoral immunity of broilers compared to control. Another study [67] reported that lincomycin induced non-significant increases in the immunity parameters of broilers.

The *IGF-1* gene is protein-encoded and produced by the liver under the stimulatory effect of growth hormone. It stimulates systemic body growth and has anabolic effects on different cells in the body, especially skeletal muscle, cartilage, bone, liver, kidney, and lung cells. In addition to the insulin-like activity, the *IGF-1* gene can regulate cellular DNA synthesis [92]. In this study, a combination of SB and lincomycin achieved the highest upregulation of *IGF-1 m-RNA* in the breast muscle of T4 and T5 birds and the highest upregulation in the thigh muscle of the T5 group. These results come per the current results of breast and thigh relative weights. Formerly, the supplementation of broilers with SB was reported to increase the expression level of the *IGF-1* gene [17]. The *TLR4* gene is encoded by the *TLR4* protein (a member of the toll-like receptor family). Its activation leads to an intracellular signaling pathway and inflammatory cytokine production, which stimulates the innate immune system [93]. In our study, the transcript level of the *TLR4* gene in liver tissue was increased in T3, T4, and T5, while it was the highest in the T5 group. Smith et al. [94] reported the upregulation of *TLR4 m-RNA* in birds fed butyrate in their diet.

In the current study, the combination of SB and lincomycin at half their recommended doses significantly lowered the caecal bacterial and clostridial counts. These findings did not apply to litter. These results agreed with previous work that displayed that micro-encapsulated butyrate diminished the pathogen colonization in caeca [95]. Notably, in a former feeding trial, 0.1% butyrate reduced caecal bacteria better than 0.2% butyrate, and they attributed that to the higher transcription level of the *AvBD9* gene in the caeca and caecal tonsil when supplementing birds with 0.1% butyrate compared to 0.2% butyrate [96]. They recommended the careful investigation of the optimum butyrate dosage for each animal species, as higher application rates of butyrate could be cytotoxic [74, 97, 98]. Weak organic acids diffuse into the cytoplasm of the bacterial cell, where they dissociate and rapidly drop the cytoplasm pH, causing bacterial cell death [99].

The supplementation of dietary butyrate derivatives boosted butyrate concentration in the large intestine and the number of neutrophils in the colonic lamina propria, which indicated that butyrate is a powerful promoter of neutrophil activity during infection [100]. Likewise, lincomycin disrupts the elongation of the peptide chain and genetic coding of the bacterial cells [101]. But antibiotics reduce the butyrate-producing bacteria in the colon, which may impair the epithelial barrier and increase susceptibility to pathogens [102]. Guinan et al. [102] documented an antibiotic-induced decline in SCFA levels in mice caeca accompanied by enriched growth and colonization of *C*. *albicans*. Those reports could interpret the significantly higher caecal bacterial and clostridial counts in lincomycin-supplemented birds T3 compared to T4, where birds received a diet with partial substitution of lincomycin with SB.

Regarding litter quality parameters, the combined dietary supplementation with SB and lincomycin lowered the litter’s moisture and nitrogen contents in T4 and T5. Previous studies pointed out that SB reduced intestinal pH and increased the activity of digestive enzymes yet increasing the digestibility and absorption of protein and minerals [74–77]. Dietary supplementation of broiler rations with SB and lincomycin could improve the birds’ ability to utilize nutrients and reduce their levels of excreta hence reducing ammonia emissions in the environment. Butyrate was indirectly associated with improving urea recycling and nitrogen retention based on the augmented expression of urea transporter in the rumen epithelia of steers delivered a rumen butyrate-enhancing diet [103].

In the current study, SB supplementation significantly increased the villi length and crypt depth of the duodenum and villi length of the ileum. These findings correlated with earlier experiments that reported improved villus length and crypt depth in the duodenum by 0.2, 0.4, and 0.6% dietary butyrate concentrations [81]. Similar results reported higher crypt depth in the duodenum of broiler chicks fed 0.2% butyrate [72]. Contrariwise, previous research [104] stated that coated SB supplementation resulted in a significant increase in villi height of jejunum compared to the control group. Similarly, another investigation [70] documented that supplementation of SB in broilers improved jejunal and duodenal histomorphometrics compared to the control. Those results suggested the increased intestinal absorption area due to the encouraging villus height growth induced by organic acid supplementation [19]. In the current work, the combination of SB and lincomycin supplementation resulted in a significant increase in villi length and crypt depth of the three intestinal regions compared to the control group, leading to high intestinal absorption and muscular weight gain. The increased villi height and villus/crypt ratios indicated the increased turnover of intestinal epithelial cells and the stimulated blood circulation of the intestine [9, 105]. On the contrary, our results disagreed with [106], who stated that lincomycin-supplemented broilers revealed necrosis of intestinal villi tips and massive inflammatory cell infiltration in intestinal propria and submucosa.

## Conclusion

Combined dietary supplementation with buffered SB and lincomycin (T4 and T5) significantly enhanced body weights, weight gains, FCR, profitability index, and carcass yields. SB supplementation (in T4 and T5) mitigated the antibiotic-induced adverse effects of lincomycin on the intestine, liver, and kidney, which appeared in lowered caecal bacterial counts, improved intestinal histomorphology, and enhanced blood biochemistry indices compared to T3. *IGF-1* and *TLR4* genes exhibited the highest expression levels in SB + lincomycin-supplemented groups (T4 and T5). Caecal bacterial and clostridial counts were the lowest in T4. Litter hygiene became more satisfactory in T4 and T5 than in other groups. Dietary SB + lincomycin (T4 and T5) increased the villi length of the intestinal mucosa. Hence, supplementing broilers’ diets by SB with lincomycin in half their doses (0.5 kg and 50 g per ton of feed, respectively) positively impacted birds’ performance and functional indices.

## Data Availability

The datasets generated during and/or analyzed during the current study are available from the corresponding author on reasonable request.

## References

[1] Tang S, Zhang S, Zhong R, Su D, Xia B, Liu L (2021). Time-course alterations of gut microbiota and short-chain fatty acids after short-term lincomycin exposure in young swine. Appl Microbiol Biotechnol.

[2] Wan F, Deng FL, Chen L, Zhong RQ, Wang MY, Yi B (2022). Long-term chemically protected sodium butyrate supplementation in broilers as an antibiotic alternative to dynamically modulate gut microbiota. Poult Sci.

[3] Makled MN, Abouelezz KF, Gad-Elkareem AE, Sayed AM (2019). Comparative influence of dietary probiotic, yoghurt, and sodium butyrate on growth performance, intestinal microbiota, blood hematology, and immune response of meat-type chickens. Trop Anim Health Prod.

[4] Ramirez J, Guarner F, Bustos Fernandez L, Maruy A, Sdepanian VL, Cohen H (2020). Antibiotics as major disruptors of gut microbiota. Front Cell Infect Microbiol.

[5] Zhang S, Zhong R, Han H, Yi B, Yin J, Chen L (2020). Short-term lincomycin exposure depletion of murine microbiota affects short-chain fatty acids and intestinal morphology and immunity. Antibiotics.

[6] Azeem S, Muneer MA, Ahmad L, Akhtar S, Pasha TN (2022). Effects of antibiotics on growth performance, immune response, and intestinal microflora of broilers. Pak J Zool.

[7] Khan EA, Ma J, Xiaobin M, Jie Y, Mengyue L, Hong L (2022). Safety evaluation study of lincomycin and spectinomycin hydrochloride intramuscular injection in chickens. Toxicol Rep.

[8] Romick-Rosendale LE, Haslam DB, Lane A, Denson L, Lake K, Wilkey A (2018). Antibiotic exposure and reduced short chain fatty acid production after hematopoietic stem cell transplant. Biol Blood Marrow Transplant.

[9] Deepa K, Purushothaman MR, Vasanthakumar P, Sivakumar K (2018). Butyric acid as an antibiotic substitute for broiler chicken–A review. Adv Anim Vet Sci.

[10] Lan R, Zhao Z, Li S, An L (2020). Sodium butyrate as an effective feed additive to improve performance, liver function, and meat quality in broilers under hot climatic conditions. Poult Sci.

[11] Bedford A, Gong J (2018). Implications of butyrate and its derivatives for gut health and animal production. Anim Nutr.

[12] Guilloteau P, Martin L, Eeckhaut V, Ducatelle R, Zabielski R, Van Immerseel F (2010). From the gut to the peripheral tissues: the multiple effects of butyrate. Nutr Res Rev.

[13] Wu W, Xiao Z, An W, Dong Y, Zhang B (2018). Dietary sodium butyrate improves intestinal development and function by modulating the microbial community in broilers. PLoS ONE.

[14] Sengupta S, Muir JG, Gibson PR (2006). Does butyrate protect from colorectal cancer?. J Gastroenterol Hepatol.

[15] Song CH, Liu ZQ, Huang S, Zheng PY, Yang PC (2012). Probiotics promote endocytic allergen degradation in gut epithelial cells. Biochem Biophys Res Commun.

[16] Tomosada Y, Villena J, Murata K, Chiba E, Shimazu T, Aso H (2013). Immunoregulatory effect of *Bifidobacteria* strains in porcine intestinal epithelial cells through modulation of ubiquitin-editing enzyme A20 expression. PLoS ONE.

[17] Eshak MG, Elmenawey MA, Atta A, Gharib HB, Shalaby B, Awaad MH (2016). The efficacy of Na-butyrate encapsulated in palm fat on performance of broilers infected with necrotic enteritis with gene expression analysis. Vet World.

[18] Peng L, He Z, Chen W, Holzman IR, Lin J (2007). Effects of butyrate on intestinal barrier function in a Caco-2 cell monolayer model of intestinal barrier. Pediatr Res.

[19] Abdelqader A, Al-Fataftah AR (2016). Effect of dietary butyric acid on performance, intestinal morphology, microflora composition and intestinal recovery of heat-stressed broilers. Livest Sci.

[20] Qaisrani SN, Van Krimpen MM, Kwakkel RP, Verstegen MW, Hendriks WH (2015). Diet structure, butyric acid, and fermentable carbohydrates influence growth performance, gut morphology, and cecal fermentation characteristics in broilers. Poult Sci.

[21] Bortoluzzi C, Pedroso AA, Mallo JJ, Puyalto M, Kim WK, Applegate TJ (2017). Sodium butyrate improved performance while modulating the cecal microbiota and regulating the expression of intestinal immune-related genes of broiler chickens. Poult Sci.

[22] Liu W, Yang Y, Zhang J, Gatlin DM, Ringø E, Zhou Z (2014). Effects of dietary microencapsulated sodium butyrate on growth, intestinal mucosal morphology, immune response and adhesive bacteria in juvenile common carp (*Cyprinus carpio*) pre-fed with or without oxidised oil. Br J Nutr.

[23] Zhang T, Xia M, Zhan Q, Zhou Q, Lu G, An F (2015). Sodium butyrate reduces organ injuries in mice with severe acute pancreatitis through inhibiting HMGB1 expression. Dig Dis Sci.

[24] Walia K, Argüello H, Lynch H, Leonard FC, Grant J, Yearsley D (2016). Effect of feeding sodium butyrate in the late finishing period on *Salmonella* carriage, seroprevalence, and growth of finishing pigs. Prev Vet Med.

[25] Song B, Li H, Wu Y, Zhen W, Wang Z, Xia Z (2017). Effect of microencapsulated sodium butyrate dietary supplementation on growth performance and intestinal barrier function of broiler chickens infected with necrotic enteritis. Anim Feed Sci Technol.

[26] Jankowski J, Juśkiewicz J, Lichtorowicz K, Zduńczyk Z (2012). Effects of the dietary level and source of sodium on growth performance, gastrointestinal digestion and meat characteristics in turkeys. Anim Feed Sci Technol.

[27] Aviagen. Ross broiler management manual, 2009. ROSS: Richmond, VA, USA., 2014; 9, 350–364.

[28] Greene ES, Maynard C, Owens CM, Meullenet JF, Dridi S (2021). Effects of herbal adaptogen feed-additive on growth performance, carcass parameters, and muscle amino acid profile in heat-stressed modern broilers. Front Physiol.

[29] Zarghi H, Golian A, Tabatabaei Yazdi F (2020). Effect of dietary sulphur amino acid levels and guanidinoacetic acid supplementation on performance, carcase yield and energetic molecular metabolites in broiler chickens fed wheat-soy diets. Ital J Anim Sci.

[30] Ahmed SA, Ahmed EA, El Iraqi KG (2018). Effect of different stocking densities as an environmental stressing factor on broiler behavior and performance. Benha Vet Med J.

[31] Khalil F, Ibrahim RR, Emeash H, Hassan A (2021). Probiotic supplementation alleviated stress and improved performance, meat quality, sensory acceptability and microbiological status of broilers. J Adv Vet Res.

[32] Elleithy EMM, Bawish BM, Kamel S, Ismael E, Bashir DW, Hamza D (2023). Influence of dietary *Bacillus coagulans* and/or *Bacillus licheniformis*-based probiotics on performance, gut health, gene expression, and litter quality of broiler chickens. Trop Anim Health Prod.

[33] Murwani R, Kusumanti E, Naumova EN (2022). *Areca catechu L*. and *Anredera cordifolia (ten) Steenis* supplementation reduces faecal parasites and improves caecal histopathology in laying hens. Int J Vet Sci Med.

[34] Biswas A, Mohan N, Raza M, Mir NA, Mandal A (2019). Production performance, immune response and blood biochemical parameters in broiler chickens fed diet incorporated with prebiotics. J Anim Physiol Anim Nutr.

[35] Toghyani M, Toghyani M, Gheisari A, Ghalamkari G, Eghbalsaied S (2011). Evaluation of cinnamon and garlic as antibiotic growth promoter substitutions on performance, immune responses, serum biochemical and haematological parameters in broiler chicks. Livest Sci.

[36] Tietz NW (1990). Clinical guide to laboratory tests.

[37] Tietz NW. Fundamentals of Clinical Chemistry. 2nd ed. NW Tietz, editor,; 1994. p. 692.

[38] Bucolo G, David H (1973). Quantitative determination of serum triglycerides by the use of the enzymes. Clin Chem.

[39] Ellefson RD, Caraway WT. Fundamentals of clinical chemistry. Ed Tietz NW. 1976; 506.

[40] Barham D, Trinder P, Analyst. 1972; 97, 142. 10.1039/an9729700142 5037807

[41] Breuer J (1996). Report on the symposium drug effects in clinical chemistry methods. Eur J Clin Chem Clin Biochem.

[42] World Organisation for Animal Health (WOAH). Newcastle disease (infection with Newcastle disease virus) (version adopted in May 2021). Chapter 3.3.14. Manual of Diagnostic Tests and Vaccines for Terrestrial Animals, twelfth edition 2023.

[43] Morgan AM, Hassanen EI, Ogaly HA, Al Dulmani SA, Al-Zahrani FA, Galal MK (2021). The ameliorative effect of N‐acetylcysteine against penconazole induced neurodegenerative and neuroinflammatory disorders in rats. J Biochem Mol Toxicol.

[44] Kasas AE, Farag IM, Darwish HR, Soliman YA, Nagar EE, Ibrahim MA (2022). Molecular characterization of alpha subunit 1 of sodium pump (*ATP1A1*) gene in *Camelus dromedarius*: its differential tissue expression potentially interprets the role in osmoregulation. Mol Biol Rep.

[45] Hassan N, Mostafa I, Elhady MA, Ibrahim MA, Amer H (2022). Effects of probiotic feed additives (biosol and Zemos) on growth and related genes in broiler chickens. Ital J Anim Sci.

[46] Kamel S, Ibrahim M, Awad ET, El-Hindi HM, Abdel-Aziz SA (2018). Differential expression of CYP2j2 gene and protein in *Camelus dromedarius*. J Biol Regul Homeost Agents.

[47] Koressaar T, Remm M (2007). Enhancements and modifications of primer design program Primer3. Bioinformatics.

[48] Untergasser A, Cutcutache I, Koressaar T, Ye J, Faircloth BC, Remm M (2012). Primer3—new capabilities and interfaces. Nucleic Acids Res.

[49] Kõressaar T, Lepamets M, Kaplinski L, Raime K, Andreson R, Remm M (2018). Primer3_masker: integrating masking of template sequence with primer design software. Bioinformatics.

[50] Dumas MD, Polson SW, Ritter D, Ravel J, Gelb J, Morgan R (2011). Impacts of poultry house environment on poultry litter bacterial community composition. PLoS ONE.

[51] Lopes M, Roll VF, Leite FL, Dai Prá MA, Xavier EG, Heres T (2013). Quicklime treatment and stirring of different poultry litter substrates for reducing pathogenic bacteria counts. Poult Sci.

[52] Esmaeilipour O, Moravej H, Shivazad M, Rezaian M, Aminzadeh S, Van Krimpen MM (2012). Effects of diet acidification and xylanase supplementation on performance, nutrient digestibility, duodenal histology and gut microflora of broilers fed wheat based diet. Br Poult Sci.

[53] Ghoneim NH, Hamza DA (2017). Epidemiological studies on *Clostridium perfringens* food poisoning in retail foods. Rev Sci Tech.

[54] Jackson ML (1973). Soil chemical analysis, pentice hall of India Pvt.

[55] Bancroft JD, Gamble M. Theories and practice of histological techniques. New York, London and Madrid: Churchil Livingstone. 2013;7:2768-73.

[56] Wickham H (2016). ggplot2: elegant graphics for data analysis.

[57] Kassambara A, ggpubr. ggplot2 based publication ready plots. R package version 0.4. 0. 2020;438. https://CRAN.R-project.org/package=ggpubr.

[58] Wickham H, Averick M, Bryan J, Chang W, McGowan LD, François R (2019). Welcome to the Tidyverse. J Open Source Softw.

[59] Kassambara A, rstatix. Pipe-friendly framework for basic statistical tests. 2023. https://rpkgs.datanovia.com/rstatix/.

[60] Ahsan U, Cengiz Ö, Raza I, Kuter E, Chacher MF, Iqbal Z (2016). Sodium butyrate in chicken nutrition: the dynamics of performance, gut microbiota, gut morphology, and immunity. World’s Poult Sci J.

[61] Petrolli TG, Albino LF, Rostagno HS, Gomes PC, Tavernari FD, Balbino EM (2012). Herbal extracts in diets for broilers. Rev Bras de Zootec.

[62] Hu Z, Guo Y (2007). Effects of dietary sodium butyrate supplementation on the intestinal morphological structure, absorptive function and gut flora in chickens. Anim Feed Sci Technol.

[63] Letlole BR, Damen EP, van Jansen C (2021). The effect of α-monolaurin and butyrate supplementation on broiler performance and gut health in the absence and presence of the antibiotic growth promoter zinc bacitracin. Antibiotics.

[64] Shareef M, Khaliq T, Faisal MN, Majeed W, Shahid M, Jamal M (2017). Comparative anti-bacterial activities of *Nigella Sativa* and lincomycin in the gut of broiler chicks. Matrix Sci Pharma.

[65] Sun X, McElroy A, Webb KE, Sefton AE, Novak C (2005). Broiler performance and intestinal alterations when fed drug-free diets. Poult Sci.

[66] Mohamed AG. Concurrent uses of diclazuril and lincomycin for controlling of severe necrotic enteritis in broiler chicks (Doctoral dissertation, Master Thesis, Department of Pharmacology, Faculty of Veterinary Medicine, Zagazig University, Zagazug, Egypt). 2016.

[67] Elkomy AA, Farag E, Elgharbawy EI, Elbadawy M (2019). Comparative studies on the effects of lincomycin and bacitracin on hematobiochemical and immunological parameters in broiler chickens. Int J Pharmacol Toxicol.

[68] Khan A, Nagra SS (2010). Performance of broiler chicks as influenced by feeding diets supplemented with organic acids. Indian J Poult Sci.

[69] Yang Q, Chen B, Robinson K, Belem T, Lyu W, Deng Z (2022). Butyrate in combination with forskolin alleviates necrotic enteritis, increases feed efficiency, and improves carcass composition of broilers. J Anim Sci Biotechnol.

[70] Sikandar A, Zaneb H, Younus M, Masood S, Aslam A, Khattak F (2017). Effect of sodium butyrate on performance, immune status, microarchitecture of small intestinal mucosa and lymphoid organs in broiler chickens. Asian-australas J Anim Sci.

[71] Papatsiros VG, Katsoulos PD, Koutoulis KC, Karatzia M, Dedousi A, Christodoulopoulos G. Alternatives to antibiotics for farm animals. CABI Reviews. 2014;1–5.

[72] Leeson S, Namkung H, Antongiovanni M, Lee EH (2005). Effect of butyric acid on the performance and carcass yield of broiler chickens. Poult Sci.

[73] Elnesr SS, Alagawany M, Elwan HA, Fathi MA, Farag MR (2020). Effect of sodium butyrate on intestinal health of poultry–a review. Ann Anim Sci.

[74] Le Gall M, Gallois M, Seve B, Louveau I, Holst JJ, Oswald IP (2009). Comparative effect of orally administered sodium butyrate before or after weaning on growth and several indices of gastrointestinal biology of piglets. Br J Nutr.

[75] Adil S, Banday T, Bhat GA, Mir MS, Rehman M. Effect of dietary supplementation of organic acids on performance, intestinal histomorphology, and serum biochemistry of broiler chicken. Vet Med Int. 2010; 2010: 479485. 10.4061/2010/479485 PMC2896631 20613998

[76] Zhang WH, Jiang Y, Zhu QF, Gao F, Dai SF, Chen J (2011). Sodium butyrate maintains growth performance by regulating the immune response in broiler chickens. Br Poult Sci.

[77] Khan SH, Iqbal J (2016). Recent advances in the role of organic acids in poultry nutrition. J Appl Anim Res.

[78] Levy AW, Kessler JW, Fuller L, Williams S, Mathis GF, Lumpkins B (2015). Effect of feeding an encapsulated source of butyric acid (ButiPEARL) on the performance of male Cobb broilers reared to 42 d of age. Poult Sci.

[79] Nazir A, Memon AU, Khan SH, Kuthu ZH, Rasool F, Ahmed Z (2017). Study on the effects of antibiotic (lincomycin) and feed additive (niacin) on the growth of broilers. Glob Vet.

[80] Chowdhury R, Islam KM, Khan MJ, Karim MR, Haque MN, Khatun M (2009). Effect of citric acid, avilamycin, and their combination on the performance, tibia ash, and immune status of broilers. Poult Sci.

[81] Panda AK, Rao SV, Raju MV, Sunder GS (2009). Effect of butyric acid on performance, gastrointestinal tract health and carcass characteristics in broiler chickens. Asian-Australas J Anim Sci.

[82] Raza M, Biswas A, Mir NA, Mandal AB (2019). Butyric acid as a promising alternative to antibiotic growth promoters in broiler chicken production. J Agricu Sci.

[83] Braun EJ, Sweazea KL (2008). Glucose regulation in birds. Comp Biochem Physiol B.

[84] Hossain ME, Nargis F (2016). Supplementation of organic acid blends in water improves growth, meat yield, dressing parameters and bone development of broilers. Bangladesh J Anim Sci.

[85] Mahdavi R, Torki M (2009). Study on usage period of dietary protected butyric acid on performance. J Anim Vet Adv.

[86] Tessari EN, Kobashigawa E, Cardoso AL, Ledoux DR, Rottinghaus GE, Oliveira CA (2010). Effects of aflatoxin B1 and fumonisin B1 on blood biochemical parameters in broilers. Toxins.

[87] Hassan HM, Mohamed MA, Youssef AW, Hassan ER (2010). Effect of using organic acids to substitute antibiotic growth promoters on performance and intestinal microflora of broilers. Asian-Australas J Anim Sci.

[88] Islam KM, Schuhmacher A, Aupperle H, Gropp JM (2008). Fumaric acid in broiler nutrition: a dose titration study and safety aspects. Int J Poult Sci.

[89] Elnesr S, Ropy A, Abdel-Razik A (2019). Effect of dietary sodium butyrate supplementation on growth, blood biochemistry, haematology and histomorphometry of intestine and immune organs of japanese quail. Anim.

[90] Ye M, Wei C, Khalid A, Hu Q, Yang R, Dai B (2020). Effect of *Bacillus velezensis* to substitute in-feed antibiotics on the production, blood biochemistry and egg quality indices of laying hens. BMC Vet Res.

[91] Deepa K, Purushothaman M, Vasanthakumar P, Sivakumar K (2017). Serum biochemical parameters and meat quality influenced due to supplementation of sodium butyrate in broiler chicken. Int J Livest Res.

[92] Yakar S, Rosen CJ, Beamer WG, Ackert-Bicknell CL, Wu Y, Liu JL (2002). Circulating levels of *IGF-1* directly regulate bone growth and density. J Clin Invest.

[93] Vaure C, Liu Y (2014). A comparative review of toll-like receptor 4 expression and functionality in different animal species. Front Immunol.

[94] Smith AL, Powers C, Beal R, Kaspers B, Kaiser P. Chapter 13 - the avian enteric immune system in health and disease. Pages 227–50 in Avian immunology 2014 (second edition). K. A. Schat, Kaspers B, Kaiser P, editors Academic Press, San Diego, CA.

[95] Van Immerseel F, Fievez V, De Buck J, Pasmans F, Martel A, Haesebrouck F (2004). Microencapsulated short-chain fatty acids in feed modify colonization and invasion early after infection with *Salmonella enteritidis* in young chickens. Poult Sci.

[96] Sunkara LT, Achanta M, Schreiber NB, Bommineni YR, Dai G, Jiang W (2011). Butyrate enhances disease resistance of chickens by inducing antimicrobial host defense peptide gene expression. PLoS ONE.

[97] Hamer HM, Jonkers DM, Venema K, Vanhoutvin SA, Troost FJ, Brummer RJ (2008). The role of butyrate on colonic function. Aliment Pharmacol Ther.

[98] Canani RB, Di Costanzo M, Leone L, Pedata M, Meli R, Calignano A (2011). Potential beneficial effects of butyrate in intestinal and extraintestinal diseases. World j Gastroenterol.

[99] Dibner JJ, Buttin P (2002). Use of organic acids as a model to study the impact of gut microflora on nutrition and metabolism. J Appl Poult Res.

[100] Hayashi A, Nagao-Kitamoto H, Kitamoto S, Kim CH, Kamada N (2021). The butyrate-producing bacterium *Clostridium butyricum* suppresses *Clostridioides difficile* infection via neutrophil-and antimicrobial cytokine–dependent but GPR43/109a-independent mechanisms. J Immunol.

[101] Morar M, Bhullar K, Hughes DW, Junop M, Wright GD (2009). Structure and mechanism of the lincosamide antibiotic adenylyltransferase LinB. Struct.

[102] Guinan J, Wang S, Hazbun TR, Yadav H, Thangamani S (2019). Antibiotic-induced decreases in the levels of microbial-derived short-chain fatty acids correlate with increased gastrointestinal colonization of *Candida albicans*. Sci Rep.

[103] Agarwal U, Hu Q, Baldwin RL, Bequette BJ (2015). Role of rumen butyrate in regulation of nitrogen utilization and urea nitrogen kinetics in growing sheep. J Anim Sci.

[104] Zhao H, Bai H, Deng F, Zhong R, Liu L, Chen L (2022). Chemically protected sodium butyrate improves growth performance and early development and function of small intestine in broilers as one effective substitute for antibiotics. Antibiotics.

[105] Fan YK, Croom J, Christensen VL, Black BL, Bird AR, Daniel LR (1997). Jejunal glucose uptake and oxygen consumption in turkey poults selected for rapid growth. Poult Sci.

[106] Ghazalah AA, El-Manylawi MA, Motawe HF, Khattab MS, Youssef YI (2021). Growth performance, nutrient digestibility, biochemical properties, hematological traits, and intestinal histopathology of broiler chicks fed mannan oligosaccharides. World’s Vet J.

