# Correction to: Impact of buffered sodium butyrate as a partial or total dietary alternative to lincomycin on performance, *IGF-*1 and *TLR*4 genes expression, serum indices, intestinal histomorphometry, *Clostridia*, and litter hygiene of broiler chickens

**DOI:** 10.1186/s13028-024-00730-4

**Published:** 2024-02-27

**Authors:** Basma Mohamed Bawish, Mohamed Farahat Selem Zahran, Elshaimaa Ismael, Shaimaa Kamel, Yasmine H. Ahmed, Dalia Hamza, Taha Attia, Khaled Nasr Eldin Fahmy

**Affiliations:** 1 Department of Veterinary Hygiene and Management, Faculty of Veterinary Medicine, Cairo University, 12211 Giza, PO Box 12211, Egypt; 2 Department of Pharmacology, Faculty of Veterinary Medicine, University of Sadat City, 23897 Minoufiya, Egypt; 3 Department of Biochemistry and Molecular Biology, Faculty of Veterinary Medicine, Cairo University, 12211 Giza, Egypt; 4 Department of Cytology and Histology, Faculty of Veterinary Medicine, Cairo University, 12211 Giza, Egypt; 5 Department of Zoonoses, Faculty of Veterinary Medicine, Cairo University, 12211 Giza, Egypt; 6 Department of Nutrition and Clinical Nutrition, Faculty of Veterinary Medicine, Cairo University, 12211 Giza, Egypt

**Correction to: Acta Veterinaria Scandinavica (2023) 65:44**

10.1186/s13028-023-00704-y

Following publication of the original article [1], we have been notified that the article text contains incorrect sentence.

It was: Butirex C4® (Avitech Nutrition Pvt. Ltd., India) is a novel feed additive of 54% SB coated with a physicalchemical matrix of buffer salts. It should be: Butirex C4® (Novation, Spain) is a novel feed additive of 54% SB coated with a physicalchemical matrix of buffer salts.

The original article was updated.
